# Search for the genes involved in oocyte maturation and early embryo development in the hen

**DOI:** 10.1186/1471-2164-9-110

**Published:** 2008-02-29

**Authors:** Sebastien Elis, Florence Batellier, Isabelle Couty, Sandrine Balzergue, Marie-Laure Martin-Magniette, Philippe Monget, Elisabeth Blesbois, Marina S Govoroun

**Affiliations:** 1 Physiologie de la Reproduction et des Comportements, UMR 6175 INRA-CNRS-Université F. Rabelais de Tours, Haras Nationaux, 37380 Nouzilly, France; 2 Unité de Recherche en Génomique Végétale UMR INRA 1165 – CNRS 8114 – UEVE 2 Rue Gaston Crémieux, CP 5708 91057 Every cedex, France

## Abstract

**Background:**

The initial stages of development depend on mRNA and proteins accumulated in the oocyte, and during these stages, certain genes are essential for fertilization, first cleavage and embryonic genome activation. The aim of this study was first to search for avian oocyte-specific genes using an *in silico *and a microarray approaches, then to investigate the temporal and spatial dynamics of the expression of some of these genes during follicular maturation and early embryogenesis.

**Results:**

The *in silico *approach allowed us to identify 18 chicken homologs of mouse potential oocyte genes found by digital differential display. Using the chicken Affymetrix microarray, we identified 461 genes overexpressed in granulosa cells (GCs) and 250 genes overexpressed in the germinal disc (GD) of the hen oocyte. Six genes were identified using both *in silico *and microarray approaches. Based on GO annotations, GC and GD genes were differentially involved in biological processes, reflecting different physiological destinations of these two cell layers. Finally we studied the spatial and temporal dynamics of the expression of 21 chicken genes. According to their expression patterns all these genes are involved in different stages of final follicular maturation and/or early embryogenesis in the chicken. Among them, 8 genes (*btg4*, *chkmos*, *wee*, *zpA*, *dazL*, *cvh*, *zar1 *and *ktfn) *were preferentially expressed in the maturing occyte and *cvh*, *zar1 *and *ktfn *were also highly expressed in the early embryo.

**Conclusion:**

We showed that *in silico *and Affymetrix microarray approaches were relevant and complementary in order to find new avian genes potentially involved in oocyte maturation and/or early embryo development, and allowed the discovery of new potential chicken mature oocyte and chicken granulosa cell markers for future studies. Moreover, detailed study of the expression of some of these genes revealed promising candidates for maternal effect genes in the chicken. Finally, the finding concerning the different state of rRNA compared to that of mRNA during the postovulatory period shed light on some mechanisms through which oocyte to embryo transition occurs in the hen.

## Background

The activation of molecular pathways underlying oocyte to embryo transition (OET) depends exclusively on maternal RNAs and proteins accumulated during growth of the oocyte [1]. During OET and preimplantation development in mice, the embryo becomes almost autonomous, and may gradually eliminate maternal components. Indeed, by the two cell stage, the major pathways regulated by maternal mRNA are targeted protein degradation, translational control and chromatin remodelling [2]. The recruitment of maternal mRNA for translation has long been recognized as a widespread mechanism to generate newly synthesized proteins in maturing oocytes and fertilized eggs [3]. Conversely, RNA that is no longer needed is actively degraded in the early embryo [4]. Moreover, careful regulation of proteolysis during the same period is likely to be important in oocytes, which are predominantly transcriptionally inactive and must often wait for long periods before fertilization in different species such as *Drosophila*, *Xenopus*, *Caenorhabditis *and Zebrafish [5]. Maternal transcripts that are present in the early pre-implantation embryo can be subdivided into two classes according to whether they are re-synthesized soon after embryonic genome activation or not. The first is common to the oocyte and early embryo and is replenished after activation of the zygotic genome. The second consists of oocyte-specific mRNA that is not subsequently transcribed from zygotic genes in the embryo. This class of mRNA may be detrimental to early post-fertilization development [6].

Maternal effect genes have been found in several species ranging from invertebrates to mammals. Wide screening of mutants has been performed in invertebrates as *Drosophila melanogaster *[7] and *Caenorhabditis elegans *[8] where several mutations lead to arrest of early embryo development. Although females bearing this type of mutation are viable and appear to be normal, the development and survival of their embryos are compromised [9]. Maternal effect mutations have also been described in other vertebrates such as *Danio rerio *for the *nebel *gene [10], and *Xenopus laevis *for the *af *gene [11]. Despite the fact that maternal effect mutations are well known in lower organisms, only a few examples have been reported in mammals. All of them are based on knock-out experiments and concern three murine genes, i.e. *Dnmt1*, *Hsf1 *and *Mater *[9]. *Mater *(Maternal antigen that embryos require) is a single-copy gene that is transcribed in growing oocytes. Although its transcripts are degraded during meiotic maturation, MATER protein persists into the blastocyst. Female mice lacking this 125 kDa cytoplasmic protein produce no offspring because of an embryonic block at the early cleavage stage. Thus, *Mater *is one of few documented genes for maternal effect in mammalian development [12]. *Mater *has been found in bovine models but there is no report in the literature on maternal effect genes conserved between species.

No information has been available to date on maternal effect genes in birds. However, birds represent a good model to observe progressive accumulation of mRNA in the oocyte before ovulation. The embryonic genome of a model bird, i.e. the chicken, is activated when the embryo contains 30,000–50,000 cells [13] 24 h after fertilization. Proteins and mRNA, accumulated as the chicken oocyte matures, are essential not only for fertilization and first cleavage but also for supporting a high number of embryonic cell divisions before genome activation. By comparison, the embryonic genome is activated at the 8-cell stage in bovines [14] and at the 2-cell stage in the mouse [15]. The avian oocyte consists of a large amount of yolk and a structure called the germinal disc (GD) [16]. The GD is a white plaque of about 3–4 mm diameter on the top of the oocyte. It contains the nucleus and 99% of oocyte organelles although it occupies less than 1% of the cell volume [17]. Structurally, and therefore functionally, the GD is mostly equivalent to the mammalian oocyte. The ovary of the reproductively active hen consists of small pre-hierarchical follicles and maturing preovulatory follicles showing a hierarchy according to size (F6 to F1) [18].

Only a few studies have reported on gene expression in the oocyte and during early embryo development in the chicken. The dynamics of the overall RNA profile of the chicken oocyte through different maturation stages has been described by Olzanska et al. [13,19-22]. Chicken vasa homolog protein (CVH) was hypothesized to be maternally inherited in the chicken embryo, since it has been localized in chicken oocytes and during first cleavage [23]. Another protein, Epidermal Growth Factor, was found in F2 GD and its potential role in follicular development has also been investigated [24].

Since oocyte-specific genes expressed during follicular maturation and after ovulation are potentially involved in the fertilization process and in early embryo development, and almost no information is available on these genes in birds, the aim of this study was to identify avian oocyte-specific genes and then to investigate the temporal and spatial dynamics of their expression during follicular maturation and early embryogenesis. We chose initially to focus on oocyte-specific genes because the accumulation of their transcripts in the oocyte should have greater consequences on fertilization and OET. Two different strategies were used to identify avian genes potentially involved in oocyte developmental competence. The first was based on a candidate gene approach and consisted of a search for avian homologs of murine oocyte genes, previously identified by digital differential display [25]. The second strategy involved a global transcriptomic approach based on chicken Affymetrix microarray. We report here several novel chicken genes with potential maternal effect identified using these two strategies. We also describe the spatial and temporal dynamics of the expression of some of these genes as well as some potential mechanisms in which they could be involved. We also compare chicken and murine orthologs in terms of their tissue specificity and their potential involvement in oocyte developmental competence and/or early embryogenesis.

## Results

### In silico search for chicken homologs of murine oocyte genes

Differential digital display analysis performed on murine tissues provided a list of 101 potentially oocyte-specific murine genes [25-27]. Bioinformatic analyses were performed on this list of genes in order to find potentially oocyte-specific chicken orthologs. Genes with a blast score higher than 100 were localized using mapview [28] and blatsearch [29] tools. The syntenic regions were checked: chromosome localization of murine genes and chicken homologs were compared, in order to obtain the correct chicken ortholog of mice genes. Only genes with sufficient homology or whose localization was in accordance with syntenic regions were selected. Forty-one chicken genes were eliminated because of their poor homology with murine genes and 32 other genes were eliminated because they were localized outside the syntenic regions. Among the remaining 28 chicken genes the transcript of only 18 genes could be correctly amplified using real time or classic RT-PCR, of which the detailed study of two genes *bmp15 *and *gdf9 *has previously been reported [30]. Thus 16 avian genes were finally retained (Table 1). The homology with murine genes was strong for 11 of these genes (blast score between 288 and 2149) and was weak for 5 (blast score between 104 and 132). The last five chicken genes were nevertheless considered as potential orthologs of murine genes and kept as candidate genes because of their correct localizations with respect to the syntenic region. Eleven of the selected genes were localized in the expected syntenic region (*btg4*,* chkmos*, *msh4*, *mtprd*, *mcmip*, *znfingerRIZ*,* discs5*, *trans fact 20, wee, zar1 *and *ktfn*). Three other genes were localized in the vicinity of the expected syntenic region (*dazL*, *fbox *and *mark3*). Two genes were localized in the unexpected syntenic region, but they were identified with the same name as murine genes (*zpA *and *zpC*).

**Table 1:** Accession numbers of murine sequences used and of homolog chicken sequences found. Bold text represents chicken genes whose syntenic regions are conserved with the appropriate murine homologs.

Murine genes				Chicken genes					
Accession number	Name	Localisation		Accession number	Name	tblastn score	Localisation		abbreviation
							
		Chromosome	Position (kb)				Chromosome	Position (kb)	

XM_205433.2	Oog2-like	4 E1	142170	XM_417634	similar to zinc finger protein RIZ. partial	100	21	4843	**znfingerRIZ**
XM_357175.2	TRAF-interacting protein	2A1	6081	XM_416727	similar to mtprd protein – mouse	102	1	100734	**mtprd**
AK054339.1	FBXO12A	9F2	109192	XM_419103	similar to hypothetical protein	104	2	91887	**fbox**
AY351591.1	Msh4	3H3	154505.7	XM_422549	similar to MutS homolog 4	107	8	29983	**msh4**
XM_138939.3	Speer-like	14A3	20635	XM_421604	similar to Discs. large homolog 5	132	6	12497.5	**discs5**
AK018361.1	Zfp393	4D2	116103.6	XM_422416	similar to Kruppel-like transcription factor neptune	288	8	20700.7	**ktfn**
NM_172481.1	Nalp9E	7A1	5473.6	XM_420951	similar to mast cell maturation inducible protein 1	473	5	3008	**mcmip**
BC066811.1	Btg4	9A5	51279	XM_417919	similar to p30 B9.10	599	24	5212.7	**btg4**
XM_355960.1	PAR-1Alike	7A3	11760.3	XM_421385	similar to MAP/microtubule affinity-regulating kinase 3 long isoform	643	5	47042.5	**mark3**
AY191415.1	Zar1	5	72968	XM_424318	Gallus gallus similar to zygote arrest 1	643	4	68326.1	**zar1**
XM_139155.2		14D3	71005	XM_416218	similar to transcription factor 20 isoform 1	688	1	46012.9	**transfact20**
NM_010021.2	Dazl	17B1	48475	NM_204218	deleted in azoospermia-like	863	2	33589.4	**dazL**
NM_020021.1	Mos	4A1	3798.4	M19412	Chicken c-mos proto-oncogene	955	2	110418	**chkmos**
NM_011775.2	ZP2	7F2	107432.5	XM_424608	similar to zona pellucida A	1146	6	15797.3	zpA
NM_201370.1	WEE1hu	6B1	40383	XM_425491	similar to Wee1A kinase	1218	1	54018.4	**wee**
NM_011776.1	ZP3	5G2	133429.5	D89097	zona pellucida C protein	2149	10	188.600	zpC

### Comparing oocyte and granulosa cells transcription profiles at final maturation steps using chicken Affymetrix microarrays

**Figure 1:**
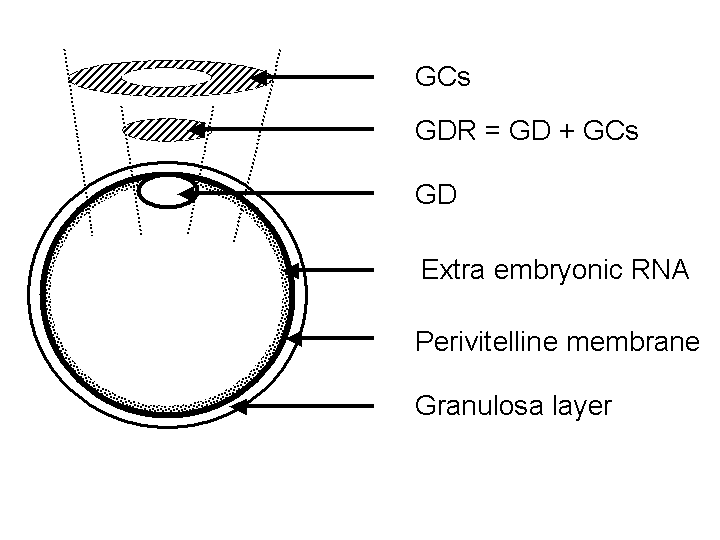
**Schematic representation of avian oocyte**. The oocyte is delimited by the perivitelline membrane, and the overlying layer of granulosa cells (GCs). Inside the oocyte, the perivitelline membrane is covered by extra-embryonic RNA. At the top of the oocyte, the germinal disc (GD) is visible. The GD with the overlying GCs constituted the germinal disc region (GDR). Two hatched area represent two different samples used in this study. The GDR comprises the GD and the lowest possible number of GCs, and GCs comprises by the GCs located in the vicinity of the GDR. Both samples are localized on the apical part of the oocyte.

**Figure 2:**
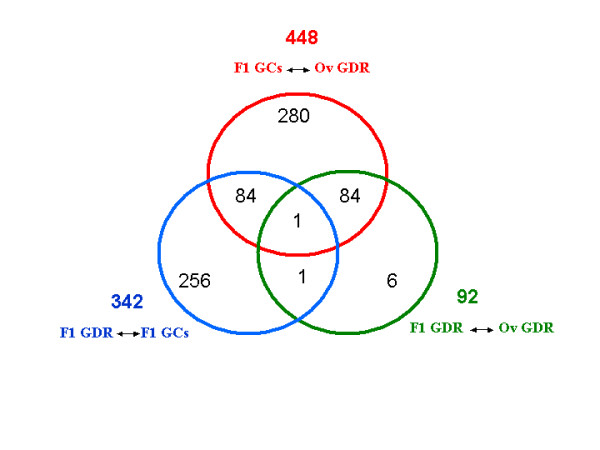
**The relationships between differentially expressed genes in different comparisons**. Diagram shows the overlap of differentially expressed genes in different comparisons. Each circle represents the total number of differentially expressed genes in one comparison. The overlapping areas represent differentially expressed genes common for different comparisons.

**Figure 3:**
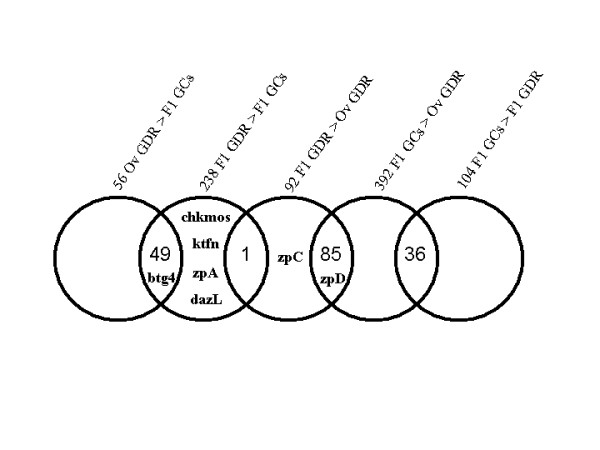
**The relationships between overexpressed genes in different comparisons**. Diagram shows the overlap of overexpressed genes in different comparisons. Each circle represents the total number of overexpressed genes in one comparison. The overlapping areas represent overexpressed genes common for different comparisons. Genes identified by the *in silico *approach and found to be overexpressed in different comparisons on the chips are indicated.

The samples studied were: F1 GCs, corresponding to granulosa cells (GCs) of the largest follicles before ovulation (F1); F1 GDR, corresponding to the germinal disc region (GDR) of F1 follicles, and Ov GDR, corresponding to the germinal disc region of ovulated oocytes (Ov) (Fig. 1). Apart from the stage of maturation the main difference between these samples involved the presence of granulosa cells. They were not present in GDR from ovulated oocytes, they were slightly present in F1 GDR, and they constituted a major component of F1 GCs (see Materials and Methods). Statistical analysis of data obtained after Affymetrix microarray hybridization provided lists of genes differentially expressed in three comparisons: F1 GDR and Ov GDR, F1 GDR and F1 GCs, and Ov GDR and F1 GCs (accession number GSE7805). There were only a few differentially expressed genes in each comparison (fewer than 500 out of 28000 genes on the Chip) (Table 2). Indeed, the first comparison, between the F1 GDR and Ov GDR, showed 92 genes over-expressed at the F1 stage, including one of our *in silico *identified genes (*zpC*). In the second comparison, between F1 GDR and F1 GCs, 342 differentially expressed genes were identified. These genes involved 104 genes over-expressed in F1 GCs and 238 genes over-expressed in F1 GDR. Five of our *in silico *identified genes *btg4*, *chkmos*, *dazL*, *zpA *and *ktfn *were found among the latter genes over-expressed in F1 GDR (Table 2). The third analysis compared the expression of genes between F1 GCs and Ov GDR. We obtained a set of 448 genes that were differentially expressed between F1 GCs and Ov GDR, of which 392 genes were over-expressed in F1 GCs and 56 genes were over-expressed in Ov GDR. We found 1 of our *in silico *identified genes among these genes, (*btg4*) (Table 2). The Venn diagram (Fig. 2) shows overlapping differentially expressed genes between different dataset comparisons. Eighty-five differentially expressed genes were common for two comparisons (F1 GCs and Ov GDR, and F1 GCs and F1 GDR). Only one gene was common for the comparisons between F1 GDR and F1 GCs, and F1 GDR and Ov GDR and 1 gene was common for all three comparisons. F1 GDR samples contained a quantity of granulosa cells in contrast to Ov GDR samples which were free of granulosa cells. Analysis of the redundancy of over-expressed genes between different comparisons was therefore performed in order to distinguish between genes found over-expressed in F1 GDR samples due to granulosa cell contamination and those really over-expressed in the oocyte (Fig. 3). This analysis revealed that 85 of 92 genes over-expressed in F1 GDR were also over-expressed in F1 GCs, both compared to Ov GDR, indicating that these genes characterized granulosa cell expression rather than variation in the expression between F1 GD and Ov GD. Thus only 7 genes should be considered as overexpressed in F1 GD compared to Ov GD. Moreover, of the 104 genes overexpressed in F1 GCs compared to F1 GDR, 36 were also overexpressed in F1 GCs compared to Ov GDR. Consequently, taking into account the latter redundancy, a total of 460 genes was overexpressed in granulosa cells compared to oocytes. On the other hand 49 genes were redundant among genes overexpressed in the Ov GDR and F1 GCs, and F1 GDR and F1 GCs comparisons.

**Table 2:** Genes differentially expressed in Affymetrix experiment. Bold text represents genes that were further studied using real time RT-PCR

Affymetrix reference	Accession number	Gene name found by Blast search	Abbreviation of genes studied	Fold change	Comparison	Number of overexpressed genes
Gga.305.1.S1_at	NM_204783.1	Gallus gallus wingless-type MMTV integration site family, member 4		150	F1 GCs and Ov GDR	56 genes overexpressed in Ov GDR
GgaAffx.13126.1.S1_at	AJ721023	Gallus gallus mRNA for hypothetical protein, clone 32l2		129		
Gga.5341.1.S1_at	BU365377	Finished cDNA, clone ChEST746h6		105		
Gga.7980.1.S1_at	CD730241	similar to Interferon regulatory factor 6		102		
Gga.4536.2.S1_a_at	BX934646.1	aldo-keto reductase family 1, member D1 (delta 4-3-ketosteroid-5-beta-reductase)		94		
Gga.9136.2.S1_a_at	CD740066	Gallus gallus tumor-associated calcium signal transducer 1		92		
GgaAffx.11525.1.S1_s_at	AJ719422	Gallus gallus claudin 1		91		
GgaAffx.20379.1.S1_at	CR524236.1	Finished cDNA, clone ChEST914o3		90		
Gga.9936.1.S1_at	BX936026.2	Amyotrophic lateral sclerosis 2 chromosomal region candidate gene protein 7		83		
Gga.5597.1.S1_at	BU450115	B-cell translocation gene 4	**btg4**	52		

Gga.14454.1.S1_at	NM_213576.1	zona pellucida protein D	**zpD**	890	F1 GCs and Ov GDR	392 genes overexpressed in F1 GCs
Gga.12391.1.S1_at	BX935169.2	Gallus gallus similar to adrenodoxin homolog		718		
Gga.1824.1.S1_at	BU350625	Gallus gallus finished cDNA, clone ChEST974b18		703		
Gga.6358.1.S1_at	BX265773	Gallus gallus similar to chromosome 9 open reading frame 61		687		
Gga.596.1.S2_at	NM_205118.1	Gallus gallus 3beta-hydroxysteroid dehydrogenase/delta5-delta4 isomerase		667		
Gga.17706.1.S1_at	CR388473.1	Gallus gallus finished cDNA, clone ChEST591g11		579		
Gga.3095.1.S1_a_at	BX932425.2	similar to hypothetical protein FLJ22662		561		
Gga.13065.1.S1_at	BU424424	Gallus gallus finished cDNA, clone ChEST537h21		559		
GgaAffx.5954.1.S1_at	ENSGALT00000015374.1	Gallus gallus similar to LRTS841		557		
GgaAffx.24194.1.S1_at	ENSGALT00000021874.1	Gallus gallus similar to CG8947-PA		534		

Gga.305.1.S1_at	NM_204783.1	Gallus gallus wingless-type MMTV integration site family, member 4		90	F1 GDR and F1 GCs	238 genes overexpressed in F1 GDR
Gga.7980.1.S1_at	CD730241	similar to Interferon regulatory factor 6		80		
Gga.6103.1.S1_x_at	BU242707	Gallus gallus similar to CG31613-PA		75		
Gga.4901.1.S1_at	BU424477	Gallus gallus similar to carbonic anhydrase 9		72		
Gga.9136.2.S1_a_at	CD740066	Gallus gallus tumor-associated calcium signal transducer 1		66		
Gga.5133.1.S1_at	NM_204218.1	Gallus gallus deleted in azoospermia-like	**dazL**	34		
Gga.5597.1.S1_at	BU450115	B-cell translocation gene 4	**btg4**	33		
Gga.8089.1.S1_at	BU258896	Gallus gallus similar to Kruppel-like transcription factor neptune	**ktfn**	18		
Gga.5714.1.S1_at	BX268842	Gallus gallus similar to zona pellucida A	**zpA**	12		
GgaAffx.9819.1.S1_at	ENSGALT00000024851.1	oocyte maturation factor Mos	**chkmos**	10		

Gga.9254.1.S1_at	CF384921			120	F1 GDR And F1 GCs	104 genes overexpressed in F1 GCs
GgaAffx.96.1.S1_s_at	ENSGALT00000000192.1	Gallus gallus similar to CG8947-PA		68		
Gga.12454.1.S1_at	BU435007	similar to relaxin 3 preproprotein		68		
Gga.11031.1.S1_at	BU450054	Gallus gallus finished cDNA, clone ChEST699k2		56		
GgaAffx.24194.1.S1_at	ENSGALT00000021874.1	Gallus gallus similar to CG8947-PA		31		
Gga.572.1.S1_at	NM_205078.1	Gallus gallus nuclear receptor subfamily 5, group A, member 2		27		
Gga.10434.1.S1_at	BX933855.1	Gallus gallus similar to chromosome 9 open reading frame 61		19		
Gga.4510.1.S1_a_at	NM_205261.1	Gallus gallus finished cDNA, clone ChEST159o8		17		
Gga.7212.1.S1_at	BU124346	Gallus gallus similar to Ephx1 protein		14		
Gga.3667.1.S1_at	NM_204839.1	Gallus gallus reversion-induced LIM protein		13		

Gga.14454.1.S1_at	NM_213576.1	Gallus gallus zona pellucida protein D	**zpD**	602	F1 GDR And Ov GDR	92 genes overexpressed in F1 GDR
Gga.12391.1.S1_at	BX935169.2	similar to adrenodoxin homolog – chicken		321		
Gga.596.1.S2_at	NM_205118.1	Gallus gallus 3beta-hydroxysteroid dehydrogenase delta5-delta4 isomerase		317		
Gga.17706.1.S1_at	CR388473.1	Finished cDNA, clone ChEST591g11		307		
GgaAffx.5954.1.S1_at	ENSGALT00000015374.1	similar to LRTS841		247		
Gga.6358.1.S1_at	BX265773	weak similarity to HUMAN Putative protein X123		240		
Gga.1824.1.S1_at	BU350625	Finished cDNA, clone ChEST974b18		213		
Gga.13065.1.S1_at	BU424424	Finished cDNA, clone ChEST738j4		195		
Gga.3095.1.S1_a_at	BX932425.2	similar to hypothetical protein FLJ22662		183		
Gga.7210.1.S1_at	NM_204389.1	Gallus gallus zona pellucida glycoprotein 3	**zpC**	64		

**Figure 4:**
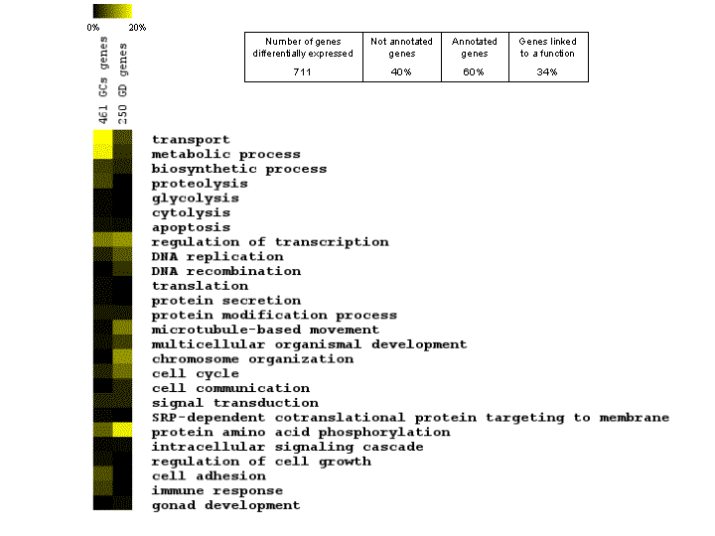
**Gene ontology (GO) classification of genes overexpressed in GDR of mature oocytes and in F1 GCs identified using Affymetrix microarray analysis**. The rows represent percentages of genes linked to a function calculated from a total number of genes linked to any function. The columns present sets of genes overexpressed in different cell layers. GO annotations were found with Netaffx software, as described in *Materials and Methods*.

Based on GO annotation the genes upregulated in GCs were mostly related to metabolic processes, transport, proteolysis, regulation of transcription, immune response and cell adhesion, whereas genes preferentially expressed in GDR were preferentially involved in cell cycle, chromosome organization, phosphorylation of proteins, regulation of transcription, multicellular organism development and DNA metabolic processes (Fig. 4).

### Tissular pattern of gene expression

**Figure 5:**
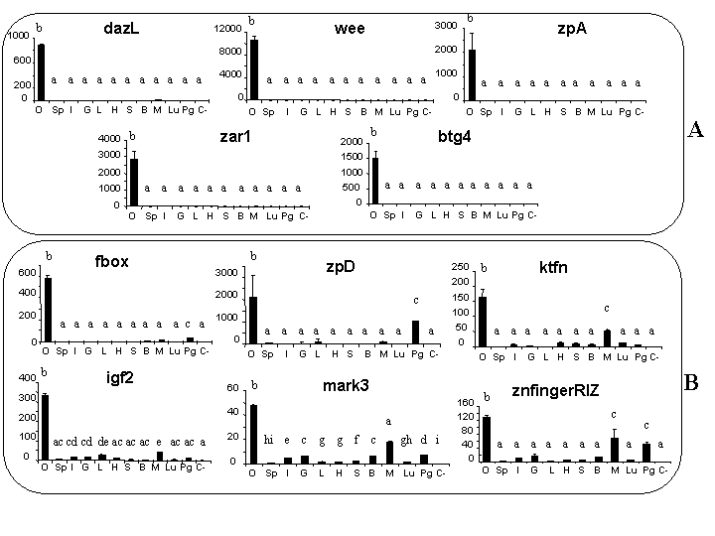
**Real-time PCR analysis of the expression of candidate genes in hen tissues**. Total RNA was isolated from whole ovary (O), spleen (Sp), intestine (I), gizzard (G), liver (L), heart (H), skin (S), brain (B), pectoralis muscle (M), lung (Lu) and pituitary gland (Pg) and real time PCR was performed as described in the *Materials and Methods*. Ribosomic RNA 18S was used as a reporter gene. The negative control (water) is indicated as (C-). The results represent the means ± SEM. The same letters indicate that differences were not significant. Different letters indicate that differences were significant (p < 0.05).

On the basis of the *in silico *and microarray approaches, 17 genes were retained for further study. Among these, 16 genes were found using the *in silico *approach as described above (Table 1), of which 6 genes (*chkmos*, *dazL*, *btg4*, *zpA*,* ktfn *and *zpC*) were also found using the microarray approache (Table 2) and 1 gene (*zpD*) was found only in the microarray approach. The latter gene was chosen because of its strong involvement in the fertilization process [31]. In contrast to *zpA *and *zpC*, the *zpD *gene was not found in the mouse [32], explaining why it was absent from the list of murine potentially oocyte-specific genes. Four other genes already known for their involvement in final follicular maturation or early embryo development in the chicken or in other vertebrates were added (*foxL2 *[33], *igf2 *[34,35], *hsf1 *[36], and *cvh *[23]) (Table 3). Finally 21 chicken genes were further studied. Real time RT-PCR performed on 11 adult tissue samples (total ovary, spleen, intestine, gizzard, liver, heart, skin, brain, pectoralis muscle, lung and pituitary gland) revealed differences in the specificity of their tissular expression patterns. Tissular expression profiles of some of these genes are presented on Fig. 4. Seven of these genes (*dazL*, *wee*, *zar1*, *zpA, btg4*, *zpC *and *chkmos*) were specifically expressed in the ovary (Fig 5A). The specificity of *zpC *and *chkmos *has previously been described and our results concerning these genes (data are not shown) were in accordance with the literature ([37] and [38], respectively). Eight genes were preferentially and strongly expressed in the ovary (Fig. 5B). Three genes from last group were slightly expressed in another tissue: *fbox *and *zpD *in the pituitary gland and *ktfn *in the muscle. We also confirmed preferential ovarian expression of *cvh *[23] and *foxL2 *[33], as well as a low expression of the latter in the pituitary gland (data not shown). Three other genes from this group (*igf2*, *mark3 *and *znfingerRIZ*) were slightly expressed in other tissues, in addition to the ovary. The last 6 genes (*trans fact 20*, *msh4*, *mtprd*, *mcmip*, *discs5 *and *hsf1*) were expressed as highly in the ovary as in other tissues (data not shown).

**Table 3:** Accession number of chicken genes selected on the basis of the literature

Bibliographic reference	Accession number	Name	Abbreviation
Govoroun et al. 2004	NM_001012612	forkhead box L2	foxL2
Aegerter et al. 2005, Heck et al. 2005	XM_421026	Insulin growth factor 2	Igf 2
Tsunekawa et al. 2000	NM_204708	Gallus gallus DEAD (Asp-Glu-Ala-Asp) box polypeptide 4 (DDX4)	cvh
Hsf1 Anckar et al. 2007	L06098	chicken heat shock factor protein 1	hsf1

### RNA state during follicular maturation and early embryo development

**Figure 6:**
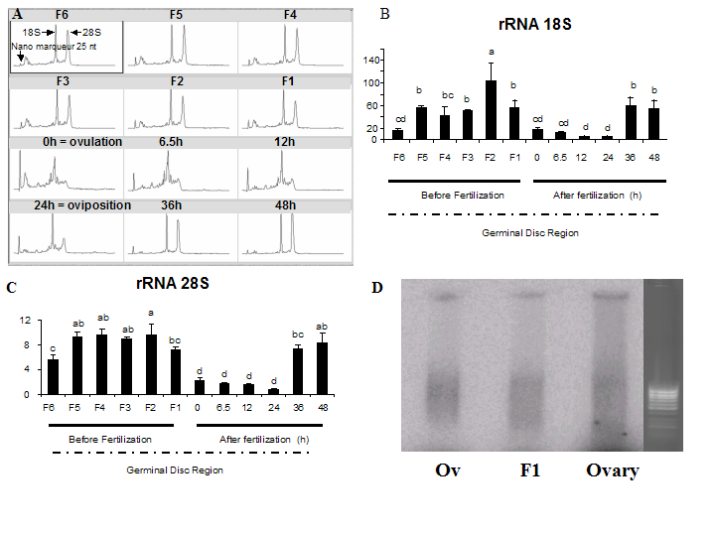
**Ribosomic and messenger RNA profiles during oocyte maturation and early embryo development**. Total RNA was extracted from the GDR of an oocyte or from the embryo. RNA quality was assessed using nanochips (Agilent technologies) as described in the *Materials and Methods*. This analysis represents rRNA 18S and 28S subunit profiles in the samples (A). Ribosomic RNA 18S and 28S subunit profiles were then confirmed by real time PCR analysis (B and C, respectively) using the TaqMan kit (Eurogentec) as described in the *Materials and Methods*. Labelled ^32^P reverse transcription was performed to investigate the quality of mRNA extracted from F1 GDR, Ov GDR and from the ovary. The radioactive signal for samples and ethidium bromide staining for the ladder are shown (D).

Analysis of total RNA, assessed with Agilent RNA nano chips (Fig. 6A), showed an atypical state of RNA from GDR between ovulation and oviposition. In our conditions, rRNA seemed degraded from ovulation until oviposition. With three different RNA extraction methods (tri reagent (Euromedex), RNeasy kit (QIAGEN) and MasterPure™ RNA Purification Kit (Epicentre Biotechnologies)), the RNA profile of GDR from the ovulation stage and from the following embryonic stages remained degraded (data not shown). Moreover, we assessed rRNA 18S and 28S by real time PCR in order to confirm this atypical RNA state (Fig. 6B and 6C). These results confirmed our previous observations; 18S and 28S rRNA expression showed a huge decrease from ovulation untill the oviposition. We then performed labelled reverse transcription to investigate whether mRNA was also degraded. We compared three samples, i.e. the RNA from GDR of F1 stage, from a whole adult ovary that had a normal rRNA profile and GDR of ovulation stage with had degraded rRNA. Electrophoresis profiles of labelled cDNA in denaturing agarose gel were almost identical for the three samples investigated and the smear corresponding to the reverse transcribed mRNA was still present in all these samples (Fig. 6D).

### Gene expression during follicular maturation and early embryo development

**Figure 7:**
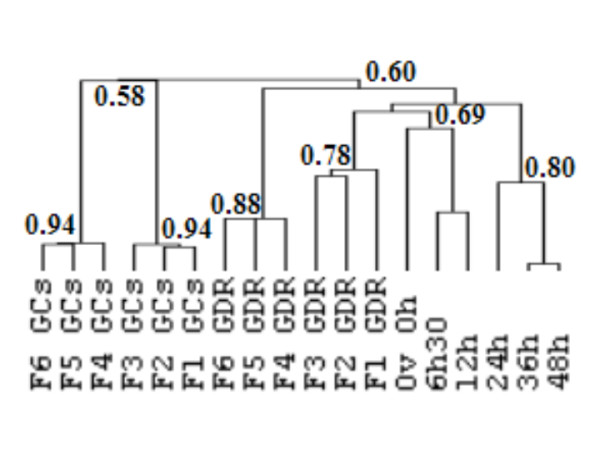
**Unsupervised hierarchical clustering of candidate genes during follicular maturation and early embryo development**. Real time PCR analysis was performed in duplicate on samples at 18 different stages with two biological replicates. Total GDR and GC RNA from different preovulatory follicles (F1 to F6), and total GDR RNA from just ovulated oocytes and early embryos at 6.5 h, 12 h, 24 h, 36 h and 48 h post ovulation were extracted as described in the *Materials and Methods*. Unsupervised hierarchical clustering of biological samples was performed using Cluster 3.0 software as described in the *Materials and Methods*. Node correlation thresholds are indicated.

A detailed study of the expression profiles during follicular maturation and early embryo development using real time RT-PCR was performed for 21 genes, for which tissue specificity of the expression was characterized. First unsupervised hierarchical clustering of our data was performed in order to confirm the biological appropriateness of the selected genes and samples (Fig 7). Two other previously studied genes (*gdf9 *and *bmp15*) were added to this analysis in order to facilitate the clustering process because of their already known oocyte-preferential localization [30]. This analysis discriminated two major groups of samples. The first included all granulosa samples from F6 to F1 (correlation threshold 0.58). The second group corresponded to GDR from F6 to the ovulation stage and to all embryo stages (correlation threshold 0.60). Several subgroups could be distinguished within each group (correlation threshold 0.69–0.94). Each subgroup corresponded to a different physiological state, indicated on Fig 6A, suggesting that the genes selected were pertinent. Moreover, unsupervised clustering arranged samples in the perfect chronological order.

**Figure 8:**
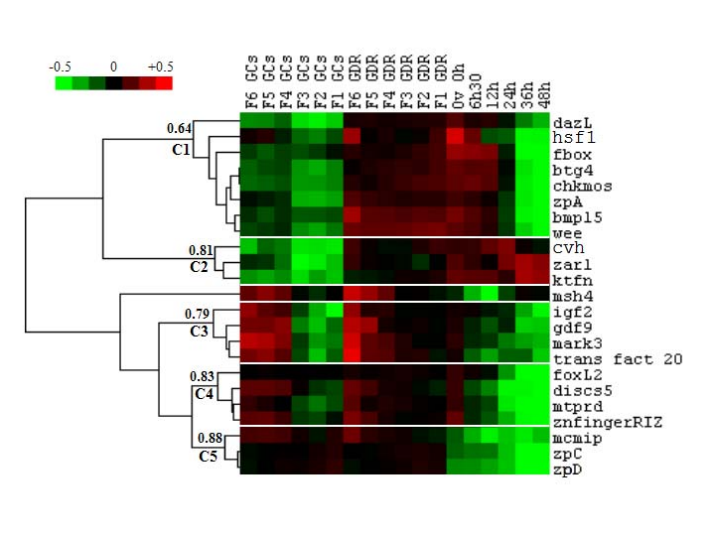
**Supervised hierarchical clustering of candidate genes during follicular maturation and early embryo development**. Real time PCR analysis was performed in duplicate on samples at 18 different stages with two biological replicates. Total GDR and GC RNA from different preovulatory follicles (F1 to F6), and total GDR RNA from just ovulated oocytes and early embryos at 6.5 h, 12 h, 24 h, 36 h and 48 h post ovulation were extracted as described in the *Materials and Methods*. Supervised hierarchical clustering of genes was performed using Cluster 3.0 software as described in the *Materials and Methods*. Five clusters are shown (C1 to C5). Node correlation thresholds are indicated for each cluster.

**Figure 9:**
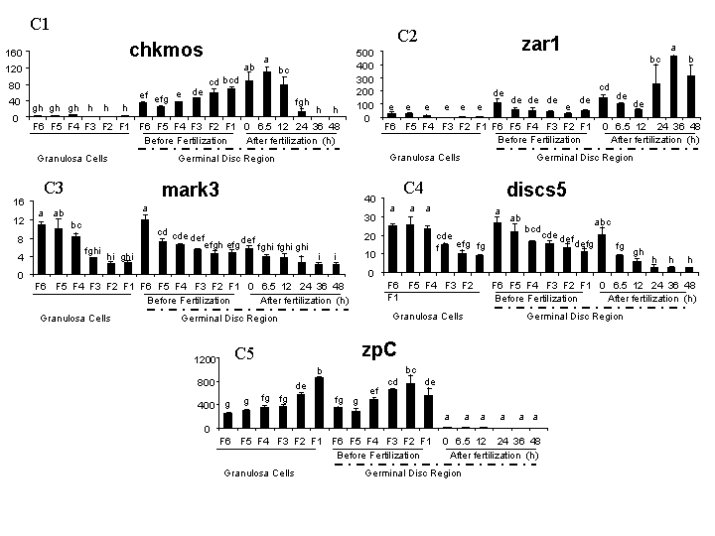
**Expression profiles of some representative genes from 5 clusters (C1 to C5), defined using supervised hierarchical clustering, during follicular maturation and early embryogenesis**. Real time PCR analyses were performed in duplicate on samples at 18 different stages with two biological replicates. Total mRNA of GDR and GCs from different preovulatory follicles (F1 to F6), and from GDR of just ovulated oocytes and early embryos at 6.5 h, 12 h, 24 h, 36 h and 48 h post ovulation were extracted as described in the *Materials and Methods*. Three different reporter genes were used (*β actin*, *ef1 α *and *gapdh*) because their expression was stable during oogenesis and early embryo development (data not shown). Results represent means ± SEM. The same letters indicate that differences were not significant. Different letters indicate that differences were significant (p < 0.05).

In order to group genes with similar expression patterns supervised clustering was performed with samples arranged first according to the nature of the sample (GCs or GDRs) and then according to chronological order (Fig. 8). Five clusters (threshold > 0.64) were identified (C1 to C5). A representative example of the expression profile characterizing each cluster is shown on Fig. 9. The graphical representation of expression profiles of all other genes with the corresponding statistical analysis of variation of their expression is supplied in additional files (see Additional file 1, 2, 3 and 4). The *chkmos*, *btg4*, *wee*, *zpA*, *dazL*, *hsf1*, *fbox *and *bmp15 *genes, forming cluster C1, were preferentially expressed in GDR. These genes showed a significant increase in mRNA expression in GDR during follicular maturation and a steady decrease after ovulation, becoming nearly undetectable at 36 h after fertilization. The c*vh*, *ktfn *and *zar1 *genes included in Cluster C2 were also preferentially expressed in GDR but, in contrast to genes from cluster C1, they were constantly expressed in the early embryo. Moreover, *ktfn *and *zar1 *genes displayed a significant increase in expression from 24 to 48 hours after fertilization. The genes from cluster C3 were expressed in GCs, in GDRs and in the embryo. Moreover, *mark3*, *igf2*, *gdf9 *and *transfact20 *genes showed a significant decrease in expression during the last stages of follicular maturation in both GDR and GCs. Cluster C4, including *discs5*, *znfingerRIZ*, *foxL2 *and *mtprd genes*, was characterized by expression that was fairly similar to that of cluster C3. The difference consisted of less pronounced variations in gene expression in GCs and more pronounced variations in gene expression in GDR for cluster C4 compared to genes in cluster C3 through the stages investigated. Genes belonging to cluster C5 were expressed in GCs and GDR but, in contrast to the clusters C3 and C4, their expression dropped dramatically at ovulation, especially for *zpC *and *zpD *whose transcripts showed increasing expression in GCs during the last stages of follicular maturation and a less marked increase in the expression in GDR. In contrast, the levels of *mcmip *decreased progressively during the same period. The last gene (*msh4*) was not clustered. Its expression profile was fairly similar to that of genes belonging to cluster C3, but its expression increased significantly 36 h after fertilization.

### Localization of gene expression in the ovary by in situ hybridization

**Figure 10:**
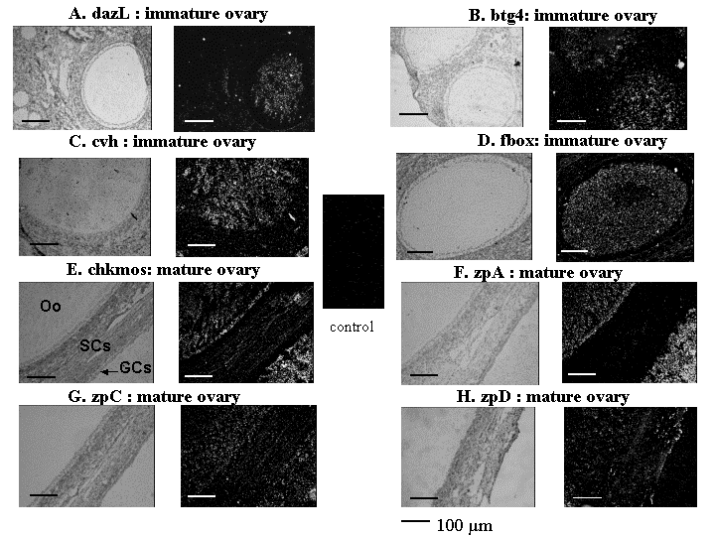
**Localization of candidate genes mRNA in the hen ovary**. Localization of *dazL*(A), *btg4 *(B), *vasa *(C), *fbox *(D), *chkmos *(E), *zpA *(F), *zpC *(G) and *zpD *(H) by *in situ *hybridization in immature ovaries (A, B, C and D) or in mature ovaries (E, F, G and H) as described in the *Materials and Methods*. Bright fields are on the left, and dark fields are on the right. The control sections were hybridized with sense probes. The hybridization with only one sense probes is represented because no signal was observed after hybridization with any sense probes. Oocytes (Oo), granulosa cells (GCs) and somatic cells (SCs) are indicated. Scale bar = 100 μm.

We performed *in situ *hybridization on mature and immature ovarian sections. All the probes were assessed on the two stages, but only the most significant results are shown on Fig. 10. For all the genes studied we detected homogeneous signals of mRNA expression in oocytes similar to those we have previously described for *bmp15 *and *gdf9 *[30]. The mRNA of four of eight genes localized by *in situ *hybridization (*btg4*, *dazL*, *cvh *and *fbox*) were detected with a high intensity signal in the oocytes from follicles of 300 μm – 600 μm from both immature (Fig. 10A,B,C and 10D) and mature (data not shown) ovaries. No significant expression was detected in somatic cells. For four other genes (*chkmos*, *zpA*, *zpC *and *zpD*) a signal was found in oocytes from the largest follicles of 500 μm-6 mm of mature ovaries (Fig. 10E,F,G and 10H). The signal was particularly high for *chkmos *and *zpA *(Fig. 10E and 10F), whereas *zpC *and especially *zpD *had weaker signals in both oocyte and somatic cells (Fig. 10G and 10H). No significant signal was detected with the corresponding sense probes.

## Discussion

In the present study we identified and characterized for the first time several genes expressed in the chicken oocyte during follicular maturation and/or in early embryo. Moreover, we showed that our candidate gene approach and microarray approach were complementary in finding new avian genes potentially involved in follicular maturation and/or early embryo development. Five genes preferentially and highly expressed in the oocyte (*btg4*, *chkmos*, *dazL*, *zpA *and *ktfn*) were identified using both microarray analysis and digital differential display on murine genes. Moreover, 2 genes (*zpC *and *zpD*), identified as overexpressed in GCs by microarray analysis, were also confirmed by real time PCR analysis to be highly preferentially expressed in chicken GCs compared to GDR. Microarray analysis identified a total of 245 genes upregulated in the hen F1 and ovulated oocytes both compared to F1 GCs. Among these, 49 overexpressed genes were common to Ov GDR and and F1 GDR, both compared to F1 GCs, and therefore represent particular interesting candidate oocyte genes for further exploration of their potential role in oocyte maturation, fertilization and OET. The fact that we found almost five times fewer genes overexpressed in the oocyte at the ovulation stage (comparison between Ov GDR and F1 GCs) than in the oocyte at F1 stage (comparison between F1 GDR and F1 GCs), compared to granulosa cells from F1 follicles, means that for some genes mRNA expression in the oocyte decreased between F1 and ovulation stages. This change in the mRNA expression levels between F1 and ovulated oocytes is probably insufficient to be detected in the comparison between Ov GDR and F1 GDR by microarray hybridization, since only 7 differentially expressed genes were identified in this comparison. On the other hand microarray analysis enabled us to detect the presence of GCs in F1 GDR samples, revealed by the redundant overexpressed genes in F1 GDR and in F1 GCs compared to Ov GDR. The functions of overexpressed genes in the various comparisons according to GO categories revealed clear differences between GCs and mature oocytes. For GCs overexpressed genes these functions were mostly related to metabolic processes, transport, proteolysis, regulation of transcription, immune response and cell adhesion, whilst for the mature oocyte they were mostly related to cell cycle, chromosome organization, phosphorylation and dephosphorylation of proteins, multicellular organism development and DNA metabolic process. These presumed functions of genes overexpressed in the oocyte are consistent with the physiological processes that it must undergo: i.e. fertilization, cleavage, chromatin remodeling, and supporting early embryo development.

The use of both bioinformatics and microarray approaches provided information on the molecular mechanisms through which OET is driven in the hen. The expression of several oocyte-specific genes increased during final follicular maturation, suggesting that transcription was still effective. After ovulation, despite the fact that 18S and 28S ribosomal RNA subunits were degraded, we showed by both labelled reverse transcription and microarray analysis that the integrity of mRNA was almost unaffected. In fact, mRNA levels were nearly the same for many genes because only 92 of 28000 genes were differentially expressed between GDRs before and after ovulation, of which only 7 really corresponded to the oocyte genes. The high number of replicates performed and the different extraction methods used strongly indicate that the difference in quality between oocyte rRNA and mRNA after ovulation is not the artifact of the experiment but reflects a real physiological feature of chicken OET, consisting probably of the arrest and degradation of the oocyte translational machinery. It could thus be hypothesized that the maternal translational system has to be replaced by the embryonic translational system. Indeed, the maternal ribosome in the embryo must be degraded before activation of the genome, in other words, before the beginning of transcription, and translation, when new embryonic ribosomes are required. This suggests that, because there are no maternal ribosomes at the stage between ovulation and oviposition, there is probably no translation or only translation of a few specific genes. If this is the case, maternal proteins should be the major essential components that support early embryo development after fertilization. This is supported by the fact that, based on GO annotation, a considerable number of the genes overexpressed in the mature oocyte are related to protein phosphorylation. Further investigation is required onto whether accumulated proteins have such an important role during these early stages of development in birds or if *de novo *protein synthesis still occurs and is dependent on the oocyte pool of ribosomes as in mammals [39].

On the basis of *in silico *and microarray approaches and analysis of the literature, 21 chicken genes were chosen in this study for further investigation of their expression using real time PCR and *in situ *hybridization. All these genes showed state-specific and/or cell type-specific expression patterns throughout the period, beginning from the first stages of final follicular maturation until embryonic genome activation. This suggests that these genes have different functions and have a role at the different physiological stages investigated. The observed decrease in the mRNA expression of almost all genes studied between ovulation and oviposition, which corresponds to late genome activation in chicken embryo [13], is consistent with a potential arrest of transcription and progressive maternal mRNA degradation occurring during meiotic maturation [40]. However, the rate of maternal mRNA degradation in the chicken seems to be considerably lower than that of rRNA, as demonstrated by the present study. Five genes (*chkmos*, *btg4*, *wee*, *dazL *and *zpA*) belonging to cluster C1 (Fig. 6B) were no longer expressed after activation of the embryonic genome, and thus transcripts of these genes are only maternally inherited. Moreover, *chkmos*, *btg4 *and *wee *genes are known to be involved in the cell cycle in other species. In our study these maternally inherited genes were increasingly expressed during follicular maturation and thus are probably used during the last stages of final follicular maturation and/or early embryo development. *Chkmos *is the chicken homolog of *mos *[38], protein kinase required for meiotic maturation in vertebrates [41,42] and for mitosis in *Xenopus laevis *[43]. Meiotic maturation is brought about by steroids using redundant pathways involving synthesis of Mos, which regulates the activity of MPF (M-phase promoting factor). The Mos-MAPK pathway has long been implicated in the arrest of mitosis in vertebrate eggs [43]. The B cell translocation gene 4 (*btg4*) belongs to a family of cell-cycle inhibitors. In the mouse and bovine, *btg4 *is preferentially expressed in the oocyte [44,45] where it exerts a marked antiproliferative activity, [46]. *Wee *is a conserved gene from invertebrates to mammals and regulates meiotic maturation during oocyte development [38,41-43]. The transcripts of *dazL *are also maternally inherited in the medaka embryo [47], and in adult medaka fish the expression of *dazL *was detected exclusively in the ovary and in the testis [48].

*ZpA*, *zpC *and *zpD*, that belong to the ZP (zona pellucida) gene family, are known to be involved in oogenesis, fertilization and preimplantation development [49]. In our study the expression pattern of *zpA *was different from that of *zpD *and *zpC *using both real time PCR analysis, where they were distributed in different clusters (C1 for *zpA *and C5 for *zpD *and *zpC *respectively), and *in situ *hybridization analysis. In contrast to chicken *zpD *and *zpC*, which were expressed in oocytes and somatic cells, chicken *zpA *was found to be specific to the oocyte, as is the case in the mouse [50,51], and expressed earlier than *zpD *and *zpC*. In the mouse the expression *of *zpA also precedes that of zp*C *[50,51]. Indeed in our *in situ *hybridization experiments *zpA *expression detected in small follicles of the mature ovary was oocyte-specific, whereas that of *zpC *and *zpD *was weaker and found in oocytes and in the somatic cells of the same follicles. Real time RT-PCR, showed increasing expression of *zpC *and *zpD *in both GCs and GDR from F6 to F1. Our finding on the dynamics of *zpC *in GCs are in accordance with a previously reported study [52]. Moreover at the F1 stage both *zpC *and *zpD *were significantly more highly expressed in GCs than in GD and this was consistent with our results for microarray hybridization, but expression decreased dramatically after ovulation. In contrast to the cellular expression pattern found for chicken *zpC*, in several mammals (murines, bovines and porcines)*zpC *(*zp3*) is specifically expressed in the oocyte. However, in the equine species, ZPC protein synthesis is completely taken over by cumulus cells [53]. These findings indicate species specificity of *zpC *distribution inside the follicle. ZPC protein plays a crucial role in the fertilization process in mammals and birds, [31,37,49].

As *zpA *other genes (*zar1, ktfn *and *cvh) *were preferentially expressed in the oocyte and might play role in fertility (*zar1, ktfn*) or in germ cell specification (*cvh*). Both *zar1 *and *ktfn *were expressed at higher levels after activation of the embryonic genome. We could therefore hypothesize that these genes might be involved not only in oocyte maturation, but also in early embryo development, just after maternally inherited genes. Of these 2 genes, only *zar1 *has been studied in reproduction. It is one of the few known oocyte-specific maternal-effect genes essential for OET in mice. In mammals and humans it is hypothesized to be involved in the initiation of embryo development and fertility control [54,55]. CVH protein has been previously proposed to be a part of the mechanism for germ cell specification in birds [16,23]. Our results concerning the spatio-temporal expression of *cvh *mRNA during follicular maturation and early embryo development are consistent with previously reported studies on the CVH protein.

The genes belonging to clusters C3 and C4 were all preferentially expressed in the ovary in both GCs and GDR and had quite similar expression patterns. Except for *foxL2*, their expression declined during follicular maturation in GCs and less in GDR and persisted at low levels in the early embryo. This suggests that they are especially involved in the first stages of final follicular maturation as well as in oocyte maturation. The chicken homolog of the mouse *par-1a-like *gene, i.e. *mark3*, is required for oocyte differentiation and microtubule organization in the Drosophila [56], and its role in cell polarity and Wnt signaling is conserved from invertebrates to mammals [57,58]. The expression of another gene (*msh4*), which did not belong to any cluster, also decreased during follicular maturation in GCs and in GD but, as for *zar1 *and *ktfn*, it showed a significant increase after embryonic genome activation. *Msh4 *is known to be involved in mediating recombination of homologous chromosomes and DNA mismatch repair in the mouse [59]. These events occur during the meiotic prophase, the stage where oocytes are blocked for a long time before meiotic maturation.

## Conclusion

In conclusion, the findings of the present study on spatio-temporal expression of 8 chicken oocyte specific genes (*chkmos*, *btg4*, *wee, zpA, dazL*, *cvh*,* zar1 *and *ktfn*) were consistent with our hypothesis that oocyte-specific genes in the chicken should play a major role in oocyte maturation, fertilization and early embryo development as in the mouse [12]. Other genes, whose mRNA expression were found in our study to be more specific for GCs or detected in both GCs and GDR depending on the stage, seem to be involved in follicular maturation (*foxl2*, *transfac 20*, *mark3*, ...) and fertilization (*zpD *and *zpC*) rather than in early embryo development. Moreover, the microarray approach provided allowed the discovery of a set of new potential chicken mature oocyte and chicken granulosa cell markers for future studies. Interestingly, 40% of these genes had no homologs in the gene databases and some of them probably correspond to specific chicken mechanisms such as hierarchical follicular maturation or rapid yolk accumulation.

## Methods

### Animals

Laying breed hens aged 60–70 weeks (ISA Brown, egg layer type, Institut de Selection Animale, Saint Brieuc, France) were housed individually in laying batteries with free access to feed and water and were exposed to a 15L:9D photoperiod, with lights-on at 8.00 pm. Individual laying patterns were monitored daily. For *in situ *hybridization, these hens and younger ones of (10 weeks old) were used to provide mature and immature ovaries, in order to study follicles at each stage. Hens used to provide fertilized eggs were bred in the same conditions and inseminated once a week.

### Collection of tissues, oocytes and embryos

Hens aged 60 weeks were used. Tissue samples were collected from the ovary, spleen, intestine, gizzard, liver, heart, skin, brain, pectoralis muscle, lung and pituitary gland. Germinal disc regions (GDR) and granulosa cells (GCs) surrounding the GDR (Fig. 1) were collected from different preovulatory follicles (F1 to F6), just ovulated oocytes and early embryos at 6.5 h, 12 h, 24 h, 36 h and 48 h post ovulation. The GDR and GCs surrounding the GDR were carefully dissected in the same way under a binocular microscope using fine forceps and scissors (World Precision Instruments) as previously described for quail oocytes [60]. After washing in phosphate buffer saline (PBS, Gibco, Cergy Pontoise, France) GDR and GCs were frozen in liquid nitrogen and then were stored at -80°C until use. For the last two stages, eggs were incubated at 37.8°C for 12 and 24 h, respectively. The 6.5 h, 12 h, and 24 h stages of embryo development correspond to stages I, V and X of the Eyal-Giladi and Kochav classification, respectively [61]. The 36 h and 48 h stages correspond to stages 3 and 6 of the Hamburger-Hamilton classification, respectively [62]. During follicular maturation the germinal disc is closely associated with its overlying granulosa cells (GCs) and forms a structure called the germinal disc region (GDR) (Figure 1). The GDR from F6-F1 follicles used for these studies consisted not only of the germinal disc but also of the overlying layer of GCs, because GD and overlying GCs cannot be completely separated [60,63,64] and the number of GCs in GDR preparations could not be counted.

### Bioinformatic analysis

A differential digital display analysis has already been performed with mouse ESTs [25-27], providing a list of murine oocyte-specific genes. Using this murine gene list, we systematically searched for chicken orthologs of these genes in international public databases pubmed [65]. Blast bit scores higher than 100 were retained. Moreover, the physical localization of genes identified on chicken chromosomes was retrieved from both mapview [28] and from blat search [29]. We also verified that chicken homologs were localized in the syntenic genomic regions conserved with that of mouse species to have a better chance that true orthologs were studied with ensembl [66].

### RNA isolation and Microarray Analysis

Total RNA was extracted from GDR of F1 and ovulated oocytes, and from GCs of F1 follicles as described above. We thus had 3 samples with a biological replicate of each sample. The RNeasy Mini Kit (QIAGEN, Hilden, Germany) was used according to the manufacturer's instructions. The tissues (GDR or GC) from 25 hens were pooled for each stage investigated to obtain enough RNA for probe synthesis. Two such a pools were constituted for each sample in order to achieve two biological replicates of microarray hybridization. All RNA samples were checked for their integrity on the Agilent 2100 bioanalyzer according to Agilent Technologies guidelines (Waldbroon, Germany). Two micrograms of total RNA were reverse transcribed with the One-cycle cDNA synthesis kit (Affymetrix, Santa Clara, CA), according to the manufacturer's procedure. Clean up of the double-stranded cDNA was performed with Sample Cleanup Module (Affymetrix, Santa Clara, CA) followed by *in vitro *transcription (IVT) in the presence of biotin-labelled UTP using GeneChip^® ^IVT labelling Kit (Affymetrix). The quantity of the cRNA labelled with RiboGreen^® ^RNA Quantification Reagent (Turner Biosystems, Sunnyvale, CA) was determined after cleanup by the Sample Cleanup Module (Affymetrix). Fragmentation of 15 μg of labelled-cRNA was carried out for 35 minutes at 94°C, followed by hybridization for 16 hours at 45°C to Affymetrix GeneChip^® ^Chicken Genome Array, representing approximately 32,773 transcripts, corresponding to over different 28,000 *Gallus gallus *genes. After hybridization, the arrays were washed with 2 different buffers (stringent: 6X SSPE, 0.01% Tween-20 and non-stringent: 100 mM MES, 0.1 M [Na+], 0.01% Tween-20) and stained with a complex solution including Streptavidin R-Phycoerythrin conjugate (Invitrogen/molecular probes, Carlsbad, CA) and anti Streptavidin biotinylated antibody (Vectors laboratories, Burlingame, CA). The washing and staining steps were performed in a GeneChip^® ^Fluidics Station 450 (Affymetrix). The Affymetrix GeneChip^® ^Chicken Genome Arrays were finally scanned with the GeneChip^® ^Scanner 3000 7G piloted by the GeneChip^® ^Operating Software (GCOS).

All these steps were performed on Affymetrix equipement at INRA-URGV, Evry, France.

The .raw CEL files were imported in R software for data analysis. All raw and normalized data are available from the Gene Expression Omnibus (GEO) repository at the National Center for Biotechnology Information (NCBI) [67], accession number GSE7805. Gene Ontology annotations were performed with NetAffx.

### RNA extraction and reverse transcription

Total RNA was extracted from whole adult tissues (ovary, spleen, intestine, gizzard, liver, heart, skin, brain, pectoralis muscle, lung and pituitary gland) using Tri-reagent (Euromedex, Mundolsheim, France) according to the manufacturer's procedure. RNA quality and quantity were then assessed by using RNA nano chips and Agilent RNA 6000 nano reagents (Agilent Technologies, Waldbronn, Germany) according to the manufacturer's instructions. Samples were stored at -80°C until use. Reverse transcription (RT) was performed to test the expression of candidate genes in different tissues and at different stages of follicular maturation and embryo development using polymerase chain reactions (PCR). One microgram of total RNA extracted from tissues or GDR was digested by RQ1 DNase (Promega, Madison, WI, USA) and reverse transcribed to first-strand cDNA using Moloney Murine Leukemia Virus reverse transcriptase I with an oligo dT-random primer mix (Promega, Madison, WI, USA) according manufacturer's instructions.

Labelled RT was performed in order to assess mRNA quality in GDR of F1 stage and just ovulated oocytes. Ten μCi αP32 dCTP was added to the reverse transcription mix in order to label cDNA. Labelled cDNA was then separated on 1.2% denaturing agarose gel (50 mM NAOH, 1 mM EDTA) by electrophoresis. A storage phosphor screen (Amersham Biosciences, Bucks, UK) was placed on the gel in an exposure cassette (Amersham Biosciences, Bucks, UK). The signal was detected one hour later with a STORM 840 (Molecular Dynamics), a phosphor screen imaging system.

### Real time RT-PCR

Specific sets of primer pairs (Sigma Genosis), designed using Vecteur NTI software to amplify fragments of 21 different transcripts, are shown in Table 4. Real time PCR reactions were carried out in 25 μl containing primers at a final concentration of 150 nm of each, 5 μl of the RT reaction diluted 1/30 and qPCR Mastermix Plus for Sybr Green I (Eurogentec) according to the manufacturer's instructions. Real time PCR was performed using an ABI Prism 7000 (Applied Biosystems). After incubation at 50°C for 2 min, and 95°C for 10 min, the thermal cycling protocol was as follows: 40 cycles at 95°C for 15 sec and 60°C for 1 min. The amounts of 18S rRNA and 28S rRNA were measured in the RT reactions diluted 1/5000 using 28S rRNA and 18S rRNA control kits (Eurogentec), respectively, according to the manufacturer's instructions. PCR amplification without cDNA was performed systematically as a negative control. 18S rRNA was used as a reporter gene in the study of the mRNA tissular expression pattern, whereas *β actin*,*gapdh *and *ef1α *were used as reporter genes in the mRNA expression study during follicular maturation and early embryo development, the expression of these three genes being similar in GCs and in GDR (data not shown). In both cases (tissular expression study or temporal dynamic study) the samples were analyzed in duplicate on the same plate for a given gene. Real time RT-PCR was performed in the temporal dynamic study with two biological replicates of each sample. Melting curve analysis was systematically performed for all genes in order to verify the specificity of the PCR product. Real time PCR efficiency (E) was measured in duplicate on serial dilutions of cDNA (pool of reverse-transcribed RNA from GDR and GC samples at the same stages as used for the temporal dynamic study) for each primer pair and was calculated using the following equation: E = (10^1/slope^)^-1^. In the tissular expression study the relative amounts of gene transcripts (R) were calculated according to the equation:

**Table 4:** Oligonucleotide primer sequences

	Abbreviation	Forward	Reverse	Efficiency	Length (bp)
Real time PCR	btg4	TTGGGTGTTTTTGGGAGG	AGTGCTTCAACTGCTTCTCAGACC	1.93	187
	chkmos	TACTCGTGTGACATCGTGACTGGC	TTGCTGGCAAACATGGTGGC	2.09	177
	dazL	TACCCATTCGTCAACAACCTGC	CCCTTTGGAAACACCAGTTCTG	1.88	184
	fbox	ACCTGTGCTGGATGATGTTGACC	CAACAAGAGGTATGTGCTTTGCG	1.81	197
	hsf1	ACCCCTATTTCATCCGTGGC	AGTCCATGCTCTCCTGCTTTCC	1.80	165
	msh4	GATTCTCGGAATGGTCACACGC	AGCATCAACAAGTGGCTCCAGG	2.16	105
	mtprd	TGAAGATCAAGGTCCAACAACTGC	TTGCTTCCTGAAACCTTTGGC	1.80	180
	mcmip	CGTGATGGCAGGAGAAGAAAGC	ATGAGTGAGGAAGGATGGGTGACC	1.80	165
	zn finger RIZ	GAGCAAAAGAGTACATCAGAGG	CCATTTGATTCACCTCTTGC	1.80	89
	mark3	AGTGAAAGAACCACTGCTGATAGGC	TTGAAGCGACAGGCGTTCTTT	1.83	91
	discs5	ATGACTGTATGGTGGTTGAGACTGC	CGGCCTTTTGAAAGTATCAGGG	1.81	176
	trans fact 20	TCAAAACTCAGCACCAGCCC	GTGCAGGTTTCTCTTGTCCACC	1.92	178
	wee	GGAGAAGAGGGTGAAGACAGAAGC	TGAGCTTCCTGTGAGGAGTTGC	1.90	121
	zar1	GTTTGTTTAGGGCTCTTCCAGGG	TTTACTCGCAGCTTTCCCAGC	2.20	92
	ktfn	ACTATTTTTCTCCTCCTGCCTCTGC	AATGCCAAATACAAGCGGGG	2.05	186
	zpA	CCTTAAATCCAACAACTCCACAGC	CAGCAAAAATCCCAACAAGAGG	1.92	131
	zpC	ACTAGCTCTGCCTTCATCACACCC	GGCAGGTGATGTAGATCAGGTTCC	1.80	109
	foxL2	Govoroun et al. 2004		1.83	
	igf 2	Heck et al. 2003		1.85	
	cvh	AGTTCCTGGCATCTTTGGGC	AGCGTCCTTTGAGAACTCCTGC	2.00	131
	zpD	TCATTGAGACAGGGAGGGAAGC	TCTTCACCACCTGCTCATAGGC	1.90	102
	gapdh	TGCTGCCCAGAACATCATCC	ATCAGCAGCAGCCTTCACTACC	2.05	199
	beta actin	CAGATGTGGATCAGCAAGCAGG	TTTCATCACAGGGGTGTGGG	1.88	107
	ef1 alpha	AGC AGA CTT TGT GAC CTT GCC	TGA CAT GAG ACA GAC GGT TGC	2.00	90

*in situ *hybridization	btg4	ACGGTCTTCTTCATCACGAGGC	TCTGTAGCACCAGCCTTCATCC		729
	chkmos	CCCTGGCAAAGATGGAAAAGC	CAGAGGGTGATGGCAAAGGAGT		732
	dazL	TGTTTTTAAGTGTGCGGGCG	ACTATTACCAGACATCTGTGTGGGC		749
	fbox	TGTGTTCCTTTACCCCGATTGC	AACTGCTACACTGCTTTCAGTCAGG		794
	zpA	TCATCGCTCCTCTCTTTGTTGG	TTTGCATGTGGGATCTCTGAGC		773
	zpC	TACCGCACGCTCATCAACTACG	ATCAGCTGCAACCTCTTTCCCG		744
	cvh	AGTTCCTGGCATCTTTGGGC	ATATCGAGCATGCGGTCTGC		792
	zpD	TATTTGCTGCTGTTCTCTGCCC	TGGTGCTGCCCTTCTATCTTCA		740

$$\text{R}=\frac{{\text{E}}_{\text{gene}}^{-{\text{Ct}}_{\text{gene}}}}{{\text{E}}_{18\text{S rRNA}}^{-{\text{Ct}}_{18\text{S rRNA}}}}$$

where Ct is a cycle threshold. In the temporal dynamic study R was calculated using following equation:

$$\text{R}=\frac{{\text{E}}_{\text{gene}}^{-{\text{Ct}}_{\text{gene}}}}{\text{Mean}({\text{Rc}}_{\text{ef}1\alpha },{\text{Rc}}_{\beta \text{actin}},{\text{Rc}}_{\text{gapdh}})}$$

where Rc is corrected relative reporter gene expression calculated as explained below. In order to take into account only fold changes in the expression levels of reporter genes between samples but not the differences in the expression levels of reporter genes in the sample, the expression levels of reporter genes in each sample (sample_i_) was adjusted against the relative amount of *ef1α *in the F1GDR sample according to the equation:

$${\text{Rc}}_{\text{reporter}}={\text{E}}_{ef1\alpha }^{-{\text{Ct}}_{ef1\alpha \text{ in F1GDR}}}\times {\text{E}}_{\text{reporter}}^{\Delta {\text{Ct}}_{\text{reporter}}}$$

where $\Delta {\text{Ct}}_{\text{reporter}}={\text{Ct}}_{\text{reporter in F1GDR}}-{\text{Ct}}_{{\text{reporter in sample}}_{\text{i}}}$.

### Hierarchical clustering

The hierarchical classification of data obtained using real time RT-PCR was performed with the Cluster 3.0 program using unsupervised single linkage or supervised complete linkage clustering in order to classify biological samples or to group together genes with a similar expression pattern, respectively [68].

### In situ hybridization

Female chickens were sacrificed at different stages of sexual development. Two types of tissue were used, i.e. mature ovaries, containing follicles of different sizes (50 μm-7 mm) from 60-week-old hens (most follicles being larger than 300 μm) and immature ovaries, containing a majority of small follicles (25–500 μm) from 10-week-old hens (most follicles being smaller than 100 μm). Mature and immature ovaries were then collected and included in Tissue-Tek (Sakura Finetek Europe BV, Zoeterwoude, The Netherlands). Frozen ovaries were serially sectioned with a cryostat (thickness 10 μm) to perform *in situ *hybridization experiments using ^35^S-labeled chicken gene cRNA. The gene antisense and sense constructs used for *in situ *hybridization were generated by inserting 700 – 800 bp fragments of chicken gene cDNA into the pGEM-T vector (Promega, Madison, WI, USA), and selecting a clone with the appropriate antisense or sense orientation. The gene cDNA fragments were generated by RT-PCR from chicken ovary mRNA using forward and reverse primers (Table 4). The *in situ *hybridization was performed as previously described [69]. Hybridization specificity was assessed by comparing signals obtained with the cRNA antisense probe and the corresponding cRNA sense probe.

### Statistical analysis

Data obtained after Affymetrix microarray hybridization analysis were normalized with the gcrma algorithm [70], available in the Bioconductor package [71]. Differential analysis was performed with the *varmixt *package of R [72]. A double-sided, unpaired *t*-test was computed for each gene between the two conditions. Variance of the difference in gene expression was split between subgroups of genes with homogeneous variance [72]. The raw P values were adjusted by the Bonferroni method, which controls the Family Wise Error Rate (FWER) [73]. A gene is declared differentially expressed if the Bonferroni-corrected P-Value is less than 0.05.

All other experimental data are presented as means ± SEM. One-way analysis of variance (ANOVA) was used to test differences. If ANOVA revealed significant effects, the means were compared by Fisher's test, with P < 0.05 considered significant. Different letters indicate significant differences.

## Authors' contributions

SE performed the experiences, the sequence alignment and the microarray analysis. FB participated in the design of the study and coordinated oocytes and embryos collection. IC participated in the experiences. SB carried out the microarrays hybridization. MLMM performed the statistical analysis of microarray data. PM participated in the design of the study and provided the list of murine oocyte genes identified *in silico*. EB participated in the design of the study. MSG conceived of the study, and participated in its design and coordination. SE and MSG wrote and revised the manuscript. All authors read and approved the final manuscript.

## Supplementary Material

Additional file 1 Real time RT-PCR analysis of expression of *chkmos, btg4, wee, zpA, dazL *and *cvh *transcript during follicular maturation and early embryo development. The real time RT-PCR data provided represent the related expression of the 6 genes *chkmos, btg4, wee, zpA, dazL *and *cvh *during follicular maturation and early embryo development, in granulosa cells and in germinal disc region. Click here for file

Additional file 2 Real time RT-PCR analysis of expression of *ktfn, zar1, zpC, zpD *and *mcmip *transcript during follicular maturation and early embryo development. The real time RT-PCR data provided represent the related expression of the 5 genes *ktfn, zar1, zpC, zpD *and *mcmip *during follicular maturation and early embryo development, in granulosa cells and in germinal disc region. Click here for file

Additional file 3 Real time RT-PCR analysis of expression of *mark3, igf2, trans fact 20, znfingerRIZ, msh4 *and *foxL2 *during follicular maturation and early embryo development. The real time RT-PCR data provided represent the related expression of the 6 genes *mark3, igf2, trans fact 20, znfingerRIZ, msh4 *and *foxL2 *during follicular maturation and early embryo development, in granulosa cells and in germinal disc region. Click here for file

Additional file 4 Real time RT-PCR analysis of expression of *discs5, mtprd, fbox *and *hsf1 *during follicular maturation and early embryo development. The real time RT-PCR data provided represent the related expression of the 4 genes *discs5, mtprd, fbox *and *hsf1 *during follicular maturation and early embryo development, in granulosa cells and in germinal disc region. Related expression measured by real time RT-PCR (as described in materials and methods). Click here for file
