# Supplementary figures and images for: Search for the genes involved in oocyte maturation and early embryo development in the hen
Source: BMC Genomics. 2008 Feb 29;9:110. doi: 10.1186/1471-2164-9-110 (PMC2322995; doi:10.1186/1471-2164-9-110)

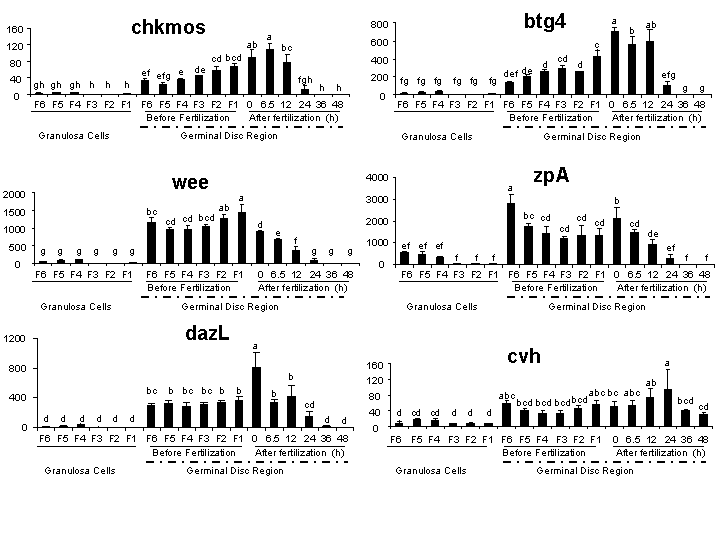

Supplement: Additional file 1 — Real time RT-PCR analysis of expression of chkmos, btg4, wee, zpA, dazL and cvh transcript during follicular maturation and early embryo development. The real time RT-PCR data provided represent the related expression of the 6 genes chkmos, btg4, wee, zpA, dazL and cvh during follicular maturation and early embryo development, in granulosa cells and in germinal disc region. [file 1471-2164-9-110-S1.png]

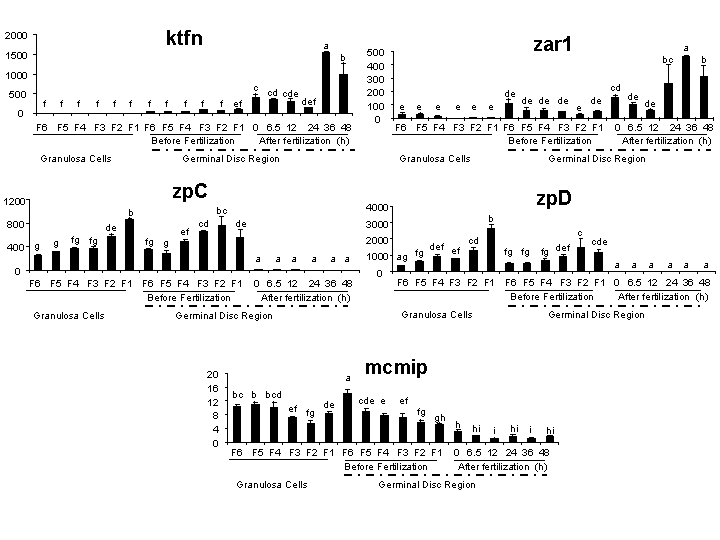

Supplement: Additional file 2 — Real time RT-PCR analysis of expression of ktfn, zar1, zpC, zpD and mcmip transcript during follicular maturation and early embryo development. The real time RT-PCR data provided represent the related expression of the 5 genes ktfn, zar1, zpC, zpD and mcmip during follicular maturation and early embryo development, in granulosa cells and in germinal disc region. [file 1471-2164-9-110-S2.png]

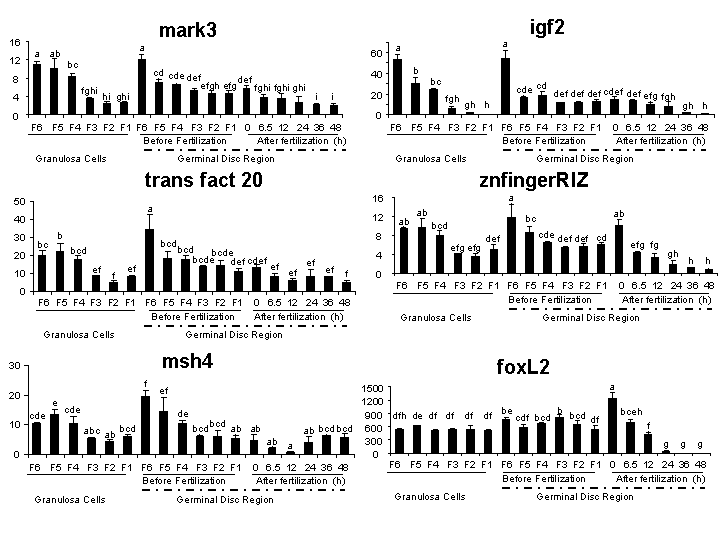

Supplement: Additional file 3 — Real time RT-PCR analysis of expression of mark3, igf2, trans fact 20, znfingerRIZ, msh4 and foxL2 during follicular maturation and early embryo development. The real time RT-PCR data provided represent the related expression of the 6 genes mark3, igf2, trans fact 20, znfingerRIZ, msh4 and foxL2 during follicular maturation and early embryo development, in granulosa cells and in germinal disc region. [file 1471-2164-9-110-S3.png]

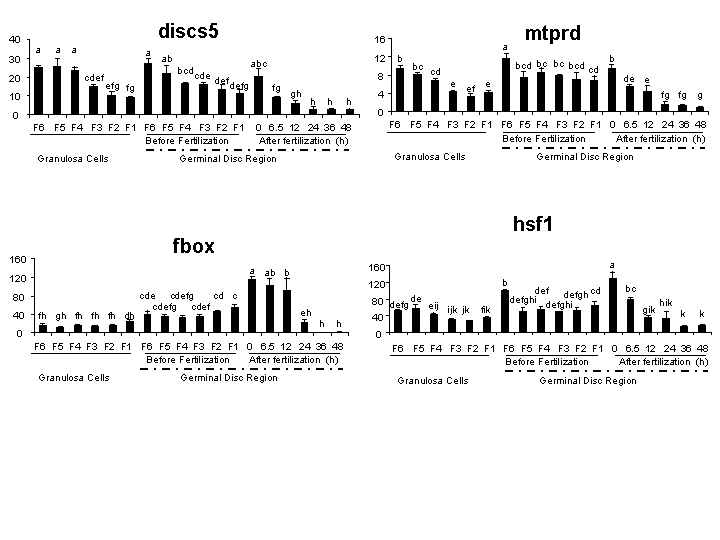

Supplement: Additional file 4 — Real time RT-PCR analysis of expression of discs5, mtprd, fbox and hsf1 during follicular maturation and early embryo development. The real time RT-PCR data provided represent the related expression of the 4 genes discs5, mtprd, fbox and hsf1 during follicular maturation and early embryo development, in granulosa cells and in germinal disc region. Related expression measured by real time RT-PCR (as described in materials and methods). [file 1471-2164-9-110-S4.png]
